# Stress Corrosion Cracking of Additively Manufactured Alloy 625

**DOI:** 10.3390/ma14206115

**Published:** 2021-10-15

**Authors:** Marina Cabrini, Sergio Lorenzi, Cristian Testa, Francesco Carugo, Tommaso Pastore, Diego Manfredi, Sara Biamino, Giulio Marchese, Simone Parizia, Fabio Scenini

**Affiliations:** 1 Department of Engineering and Applied Sciences, School of Engineering, University of Bergamo, 24044 Dalmine, Italy; sergio.lorenzi@unibg.it (S.L.); cristian.testa@guest.unibg.it (C.T.); francesco.carugo@unibg.it (F.C.); tommaso.pastore@unibg.it (T.P.); 2 Research Unit of Bergamo, National Interuniversity Consortium of Materials Science and Technology (INSTM), 24044 Dalmine, Italy; 3 Department of Applied Science and Technology, Politecnico di Torino, 10129 Torino, Italy; diego.manfredi@polito.it (D.M.); sara.biamino@polito.it (S.B.); giulio.marchese@polito.it (G.M.); simone.parizia@polito.it (S.P.); 4 Materials Performance Centre and Corrosion and Protection Centre, Department of Materials, University of Manchester, Manchester M13 9PL, UK; fabio.scenini@manchester.ac.uk

**Keywords:** laser powder bed fusion, Alloy 625, stress corrosion cracking, hydrogen embrittlement

## Abstract

Laser bed powder fusion (LPBF) is an additive manufacturing technology for the fabrication of semi-finished components directly from computer-aided design modelling, through melting and consolidation, layer upon layer, of a metallic powder, with a laser source. This manufacturing technique is particularly indicated for poor machinable alloys, such as Alloy 625. However, the unique microstructure generated could modify the resistance of the alloy to environment assisted cracking. The aim of this work was to analyze the stress corrosion cracking (SCC) and hydrogen embrittlement resistance behavior of Alloy 625 obtained by LPBF, both in as-built condition and after a standard heat treatment (grade 1). U-bend testing performed in boiling magnesium chloride at 155 and 170 °C confirmed the immunity of the alloy to SCC. However, slow strain rate tests in simulated ocean water on cathodically polarized specimens highlighted the possibility of the occurrence of hydrogen embrittlement in a specific range of strain rate and cathodic polarization. The very fine grain size and dislocation density of the thermally untreated specimens appeared to increase the hydrogen diffusion and embrittlement effect on pre-charged specimens that were deformed at the high strain rate. Conversely, heat treatment appeared to mitigate hydrogen embrittlement at high strain rates, however at the slow strain rate all the specimens showed a similar behavior.

## 1. Introduction

Additive manufacturing (AM) is a key enabling technology for the manufacture of unique geometries that are difficult to produce by means of conventional techniques, such as casting followed by milling and turning. The application of AM takes on a significant importance for maintaining and increasing competitiveness and innovation on the increasing market demand.

Laser bed powder fusion (LPBF) is an AM technology for the fabrication of semi-finished components directly from computer-aided design modelling, through melting and consolidation, layer upon layer, of a metallic powder, with a laser source. Recent studies have demonstrated that during AM the microstructure experiences complex thermal modifications (such as directional heat extraction, repeated melting and rapid solidification cycles), repeated solid-state phase transformations, and a complex microstructural and mechanical properties evolution that are not typically found in conventional processes [1].

Research in the field of metal additive manufacturing has tried to overcome all of the issues connected with the production process to develop dense, defect-free and reliable materials [2]. Post-build heat treatments are generally adopted to reduce internal stresses and increase materials performance through the modification of microstructures [3]. Stress relief is also routinely used to reduce the residual stress that is built up in thermal cycles, to avoid excessive geometrical distortion of the AM components and micro-defects that could lead to premature failure of components [4]. Homogenization heat treatments are mainly needed to dissolve undesired second phases, and to produce materials and parts with predictable and reproducible microstructure and materials’ properties [5]. However, such heat treatments can induce grain growth and affect mechanical properties such as the hardness, yield strength, tensile strength, fatigue strength, and impact strength of these alloys. A comprehensive understanding of the effect of heat treatment on the microstructure and phase evolution of AM alloys is therefore required. In fact, these unique microstructures can be more susceptible to different forms of corrosion than those of conventionally produced alloys [3,4,5,6,7,8,9,10,11,12,13,14,15,16,17,18,19,20].

Understanding the effect of manufacturing on the microstructure, and its implication on the resistance to corrosion, is fundamental to ensuring integrity during their operation in demanding environments. Alloy 625 has found widespread application in the aerospace, marine, and nuclear industries where complex shapes are often required; it is also a reference material for the oil and gas field due to its high yield strength value, fatigue resistance, and excellent corrosion behavior in very aggressive environments. Alloy 625 is a precipitation hardened alloy where very fine Ni_3_(Nb, Al, Ti) phases, which form during aging treatments, strengthen the austenitic matrix. The formation, or evolution, of the second phases during high temperature operation significantly modifies the yield strength and ductility of the alloy [21]. At the same time, since the machinability of Alloy 625 is poor, this material is an excellent candidate for additive manufacturing [22].

The resistance to pitting of Alloy 625 is very high, much higher than the traditional austenitic stainless steels. In fact, electrochemical results, already published by the present authors elsewhere, demonstrated that the alloy produced by laser powder bed fusion was not susceptible to pitting in either 1 M H_2_SO_4_ at 40 °C, or neutral or acidified NaCl 35 g/L at 40 °C, and had a crevice corrosion resistance slightly higher than that of traditional wrought material; these results were also confirmed via critical crevice temperature (CCT) tests [23,24]. Post manufacturing heat treatment can also have an impact on corrosion resistance; however, the alloy may become susceptible to intergranular corrosion if subjected to improper solubilization treatment [25]; in fact, the formation of carbides and secondary phases can affect localized corrosion resistance and environment assisted cracking susceptibility. Moreover, whilst Alloy 625 is generally immune to stress corrosion cracking (SCC) in hot chloride solution, sporadic failure has been reported [26] and it can also suffer from SSC when exposed to supercritical water [27].

Alloy 625 can also be susceptible to hydrogen embrittlement (HE) when exposed to high hydrogen pressures at high temperatures [28] or following relatively low cathodic polarizations in water solutions or in molten salts [27,28,29,30,31,32]. Murakami et al. reported that hydrogen diffusion into the alloy, and hydrogen effects after hydrogen absorption, were independent of the charging method despite the differences in adsorption and dissociation reaction on the specimen surfaces [30]. Thus, cathodic hydrogen charging can be used to study the hydrogen embrittlement phenomena in nickel alloys. Several papers outlined the effect of hydrogen embrittlement during cathodic polarization of nickel alloys tested under slow strain rate tensile (SSRT). In general, hydrogen embrittlement effects are evident for a strain rate lower than 10^−5^ s^−1^ [33,34,35]. SSRT tests conducted by Huang et al. [36] at 4 · 10^−6^ s^−1^ on wrought precipitation hardened Alloy 625 polarized at −1.1 V vs. Ag/AgCl in substitute sea water demonstrated the strong susceptibility of this alloy to HE; in fact, the elongation to failure of cathodically polarized samples decreased to 60% and the reduction of area decreased to 50% relative to the values in air. The microstructure of the alloy is also known to strongly influence the susceptibility of the precipitation hardened alloys. In fact, it is suggested that the susceptibility of alloys 718 and 625 to HE increases with the volume fraction of precipitates in the matrix and at the grain boundaries [37], and that γ” phase has a detrimental effect on hydrogen embrittlement, whilst γ’ has a beneficial effect [38].

Hydrogen embrittlement and stress corrosion cracking in nickel alloys are strongly dependent on their microstructures, which is also dependent on the heat treatment. Therefore, the aim of this work is to investigate the SCC and hydrogen embrittlement resistance of Alloy 625 obtained by LPBF in either as-built condition or after “grade 1” standard heat treatment (i.e., annealing at 980 °C for 32 min followed by water quenching), which is commonly used in the oil and gas industry [33]. In fact, the unique microstructure of the LPBF-obtained alloy could be tailored by a high temperature heat treatment which can induce recrystallization, thus leading to the formation of materials with microstructure and mechanical properties such as those expected on the traditional wrought and annealed materials.

## 2. Materials and Methods

Gas atomized Alloy 625 powder provided by EOS GmbH (Krailling, Germany) was used in this study; the compositions of the LPBF powder and on the printed metal were very close to each other (see Table 1), thus confirming the limited alloy elements sublimation during the printing process. The specimens were produced by means of an EOSINT M270 Dual mode version (EOS GmbH, Krailling, Germany). The main process parameters were a laser power of 195 W, a scanning speed of 1200 mm/s, a hatching distance of 0.09 mm and a layer thickness of 0.02 mm combined with a scanning strategy with rotation of 67° between consecutive layers. The selected parameters resulted in a high densification level of the samples close to a relative density of 100%, as reported in previous studies [23,39].

Two different metallurgical conditions were investigated: (i) un-treated alloy with the characteristic microstructure obtained at the end of LPBF (named UT-LPBF), and (ii) heat treated at 980 °C for 32 min (grade 1 [33]) followed by water quenching (named HT-LPBF specimens).

The stress corrosion cracking tests were carried out in a 1 L flask using boiling saturated magnesium chloride solution at 155 °C according to ASTM G36, and at 170 °C using ASTN G30 U-bend specimens that employed Alloy 625 fasteners. The geometry and dimension of the specimens are reported in Supplementary Figure S1. The specimens were manufactured with a flat face that was either parallel to the building direction (Z direction) or perpendicular to the building direction (XY plane) (specimen’s orientation was added in Supplementary Figure S2).

The flat specimens were bent around a curved metal mold to obtain a U-bent sample and fastened with a fitting; the surface portion of the curved part (5 mm radius in accordance with the standard) of the specimen was subjected to a stress equal to the flow stress. After bending, the specimens were left with the as-built surface. Before bending, the specimens were degreased in acetone in an ultrasound bath, and subsequently were placed into the flask with the boiling MgCl_2_ (Carlo Erba, RPA Reagents, Cornaredo, Milano, Italy) solution. The concentration of Mg_2_Cl was 45% to achieve the boiling temperature at 155 °C, however the concentration was also adjusted, as suggested by [40] to reach the boiling temperature of 170 °C. Two specimens for each SCC testing temperature were used.

The duration of the tests was 7 days, with intermediate optical observations every 24 h to verify the presence of cracks using a 50× magnification lens.

**Figure 1:**
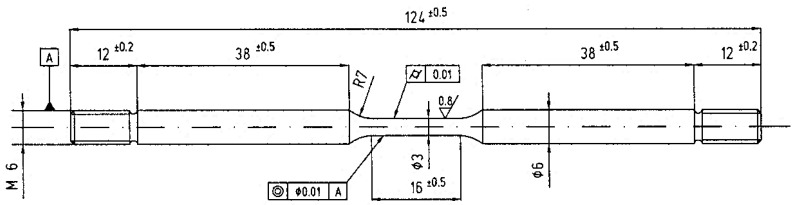
Geometry of the SSRT specimens (all values are in mm).

SSRT tests were also conducted using round dogbone samples whose geometry is schematically reported in Figure 1, by means of a homemade SSRT testing machine with four independent loading stations. The load was applied through an electric motor and reduction gears that could impose displacement rates between 5 × 10^–7^ and 5 × 10^–3^ mm/s. The calibration of the cells was performed independently. Acquisition system (spider 8 HBM Italy, Milano, Italy) recorded the load and a function of time. A strain rate of either 3.15 × 10^−6^ s^−1^ or 3.15 × 10^−5^ s^−1^ was chosen.

The cylinders used to extract the SSRT samples were built with the longitudinal axes oriented perpendicular to the building direction. The specimens were also manually ground with SiC paper up to 1200 grit in order to remove the machining marks. The tests were conducted in a glass cell with one liter of volume, using a standard Ag/AgCl (3 M KCl) electrode (Amel, Milano, Italy) as reference, and graphite and counter electrodes (Supplementary Figure S3). The tests were carried out in aerated substitute ocean water (SOW) (completed according to ASTM D1141 using reagents for analysis RPE Carlo Reagents, Cornaredo, Milano, Italy) at pH equal to 8.2. The free corrosion potential of the all the specimens was in the range of −0.255 to −0.155 V vs. Ag/AgCl. The samples were polarized at −1.1 V vs. Ag/AgCl (using 2052 AMEL, Milano, Italy, instrument), and the recorded steady state cathodic current density for all specimens was 0.085 μA/cm^2^.

For those SSRT tests carried out at a strain rate of 3.15 × 10^−6^ s^−1^, the test started immediately after polarizing the specimens and lasted about 16 h. In order to establish the effect of the strain rate on the hydrogen embrittlement susceptibility of the alloy, another test was conducted at a 10× faster strain rate (3.15 × 10^−5^ s^−1^) after the samples had been hydrogen pre-charging at the same potential (−1.1 V vs. Ag/AgCl) for 16 h.

The susceptibility to SCC was expressed in terms of the area reduction percentage (RA%) calculated by Equation (1) according to the NACE TM-0198 standard:

(1) $$RA\%=\frac{\left(D_{f}^{2}-D_{i}^{2}\right)\times 100}{D_{i}^{2}}$$

where *D_i_* and *D_f_* are respectively the initial and final diameter of the gouge length of the specimens. The hydrogen embrittlement index (*HE_index_*) was evaluated by the area reduction ratio after fracture for the specimen cathodically polarized (*RA_test_*) to the corresponding value determined in the control environment (*RA_air_*), and it was calculated according to Equation (2):

(2) $$HE_{index}=\left(1-\frac{RA_{test}}{RA_{air}}\right)\times 100$$

## 3. Results

Table 2 summarizes the results of the SCC tests: both building directions did not show presence of stress corrosion cracks at the two tested temperatures, confirming the good stress corrosion cracking resistance of Alloy 625. A small loss of mass was registered during the tests: the loss of mass was relatively scattered, and it was not possible to have a good correlation with the building direction or the heat treatment; most likely this mass loss was due to the detachment of small debrides present on the surface of the specimens, i.e., due to un-melted powder or balling.

**Figure 2:**
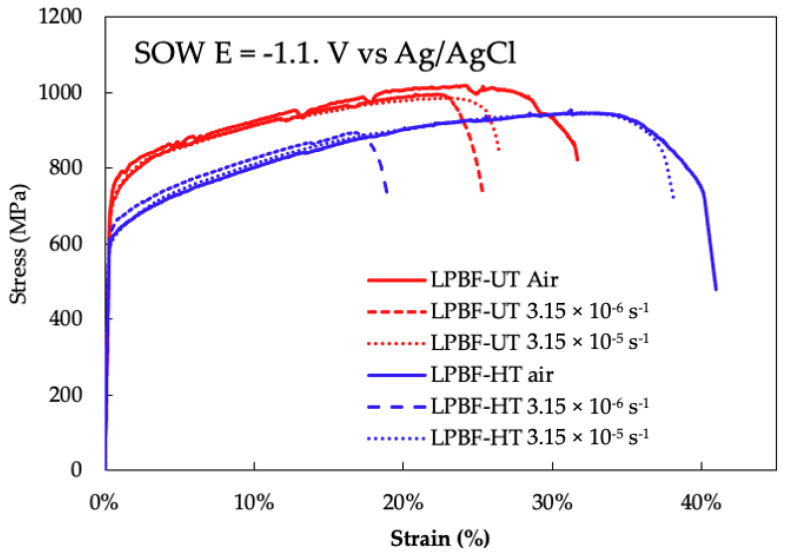
SSRT results of LPBF-UT (red line) and LPBF-HT (blue line) at 3.15 × 10^−6^ s^−1^ in air, at 3.15 10^−6^ s^−1^ in SOW under cathodic protection, and at 3.15 × 10^−5^ s^−1^ after hydrogen pre-charging.

The SSRT curves are shown in Figure 2. The UT-LPBF specimen (red curves in Figure 2) tested in air at 3.15 × 10^−6^ s^−1^ showed a very pronounced plastic region achieving 1018 MPa of ultimate stress and 32% of strain to failure. The tests in SOW were carried out at the same cathodic polarisation, −1.1 V vs. SCE, and had the same duration (about 16 h), but with two different strain rates: the first at 3.15 × 10^−6^ s^−1^ and the second at 3.15 × 10^−5^ s^−1^; in this last case the specimen was pre-charged with hydrogen before starting the tensile test. The cathodic polarised specimen tested at the lower strain rate presented a reduction of the total strain with respect to the specimen tested in air, achieving a value equal to 25%, the specimen pre-charged with hydrogen showed less reduction of ductility (26%). The heat treatment (blue curves in Figure 2) decreased the yield and maximum stress of all specimens, and increased the ductility, which in air was 61% of total strain. The differences between the reduction of ductility with respect to air are more evident in these specimens (LPBF-HT): the specimens cathodically polarised in SOW and strained at 3.15 × 10^−5^ s^−1^ presented a total elongation of 22%, whereas the pre-charged specimen showed a total strain of 50%. The reduction of area values are in agreement with the total elongation values, as summarized in Table 3.

### Fractographic Analysis

**Figure 3:**
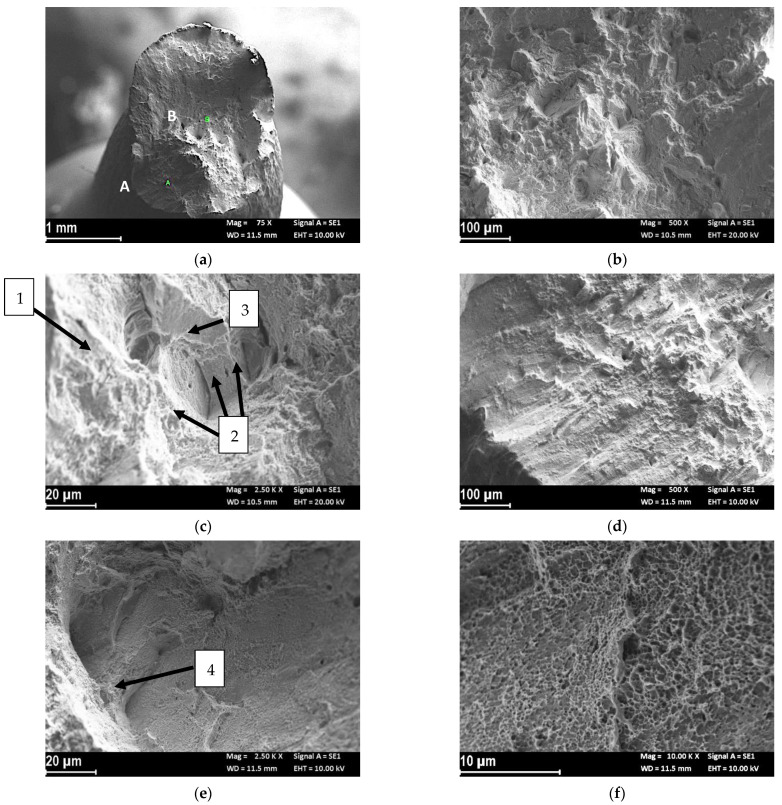
Secondary electron fractographic analysis of the UT-LPBF specimen after the SSRT test in air: (**a**) macro image; (**b**) close-up of the center of the specimen (zone B in (**a**)); (**c**) close-up at higher magnification of Figure 3b, the arrows indicate: (1) presence of large dimple associated to inclusion, (2) edge of the melt pool, and (3) low toughness zone; (**d**) close-up of zone A of (**a**) fracture zone of shearing; (**e**) close-up of (**d**) in which is evident the separation of the melt pools, (**f**) close-up of zone (4) in (**e**) in which are evident micro dimples in the separation of the melt pools.

Figure 3 shows the fracture morphology of the UT-LPBF specimen tested in air. The specimen did not have a perfectly regular cone/cup fracture surface (Figure 3a). In the center of the specimen, the fracture was characterized by irregular dimples of different sizes (zone B in Figure 3a and close up of Figure 3b,c) sometimes associated with inclusions (zone 1 in Figure 3c), or, more frequently to the border of the melt pools (zone 2 in Figure 3c). Analyzing the center of the specimen at high magnifications, areas of low toughness and points of cleavage almost appeared (zone 3 in Figure 3c). At the border of the specimen (zone A in Figure 3a) the fracture mode was due to shearing (Figure 3d). On the shearing zone there were micro-separations corresponding with either the edge of the melting pool (zone 4 in Figure 3e), or the dimples (Figure 3f). The different cracking behavior in different regions suggested that there were some zones more ductile than others in the specimen, as it was possible to observe by the sharp decrease in the stress vs. strain curves (Figure 2).

**Figure 4:**
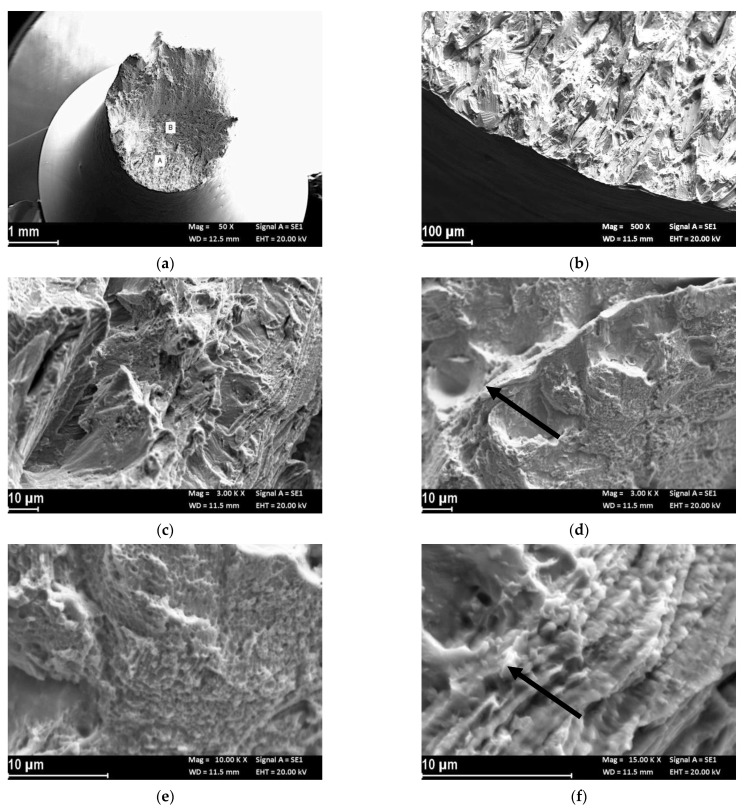
Secondary electron images of the fracture surface of UT-LPBF specimen after the SSRT test at strain rate 3.15 × 10^−6^ s^−1^ in SOW and cathodic polarization at −1.1 V vs. Ag/AgCl: (**a**) macro image; (**b**) close-up of the trigger zone (zone A in (**a**)) with brittle morphology; (**c**) close-up of (**b**) in which is evident a quasi-cleavage growth, (**d**) close-up of (**b**) in which is evident the separation of the melt pool (arrow) and presence of dimples in the upper melt pool; (**e**) close-up of the center of the specimen (zone B in (**a**)), presence of dimples and brittle zone; (**f**) close-up at higher magnification of (**e**) in which are evident the presence of intermetallic precipitates.

Figure 4a reports the macro image of the UT-LPBF specimen after the test in SOW and cathodic polarization of −1.1 V vs. Ag/AgCl. The specimen still showed plastic deformation, as can be seen from the reduction of area percentage shown in Table 3, but the fracture surface had a flat trigger area (zone A of Figure 4a), which extended to the center of the specimen (zone B of Figure 4a) and the final break was due to sliding by shearing. The trigger zone had a quasi-cleavage aspect (Figure 4b), which is typical of hydrogen embrittlement phenomena. The higher magnification details (Figure 4c) show the alternation of flat areas and small dimples. Microfractures were evident at the separations of the fusion wells, which changed the fracture propagation (Figure 4d). The fracture surface at the center of the specimen (zone B of Figure 4a) was mixed, with brittle area interspersed with small dimples (Figure 4e). The high-magnification image shows the presence of small intermetallic precipitates on the flat facets (Figure 4f).

**Figure 5:**
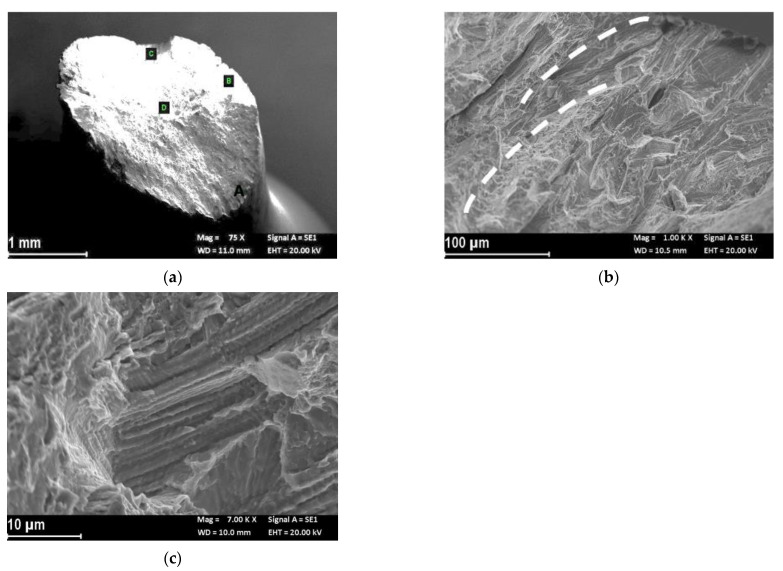
Fractographic analysis of the UT-LPBF specimen after the SSRT test at strain rate 3.15 × 10^−5^ s^−1^ in SOW and cathodic polarization at −1.1 V vs. Ag/AgCl after 16 h of hydrogen pre-charging: (**a**) macro image; (**b**) close-up of zone A, and (**c**) a similar morphology is observed in zone C; close-up of zone B; zone D in (**a**) is fully ductile.

Figure 5a shows the macro image of the fracture surface of the UT-LPBF specimen pre-charged with hydrogen for 16 h and then strained at 3.15 × 10^−5^ s^−1^. The fracture surface had some small trigger areas with a morphology typical of hydrogen embrittlement (zone A, B and C of Figure 5a; the close-up of zone A is shown in Figure 5b, the close-up of zone B is shown in Figure 5c), while the central area had a mixed fracture consisting of numerous dimples, small brittle facets and rare areas of quasi-cleavage. The final breakage of the specimen occurred by sharing. The value of the normalized reduction of area was very similar to that obtained in the previous case. At high magnification for areas A (Figure 5b), B (Figure 5c) and C of the specimen, a morphology similar to that observed in Figure 4 could be noted, with many brittle facets arranged perpendicularly to the direction of propagation of the fracture (along the dendritic growth) that occasionally jumped on a different plane, and a separation of the border of the melting pool (dotted line in Figure 5b).

**Figure 6:**
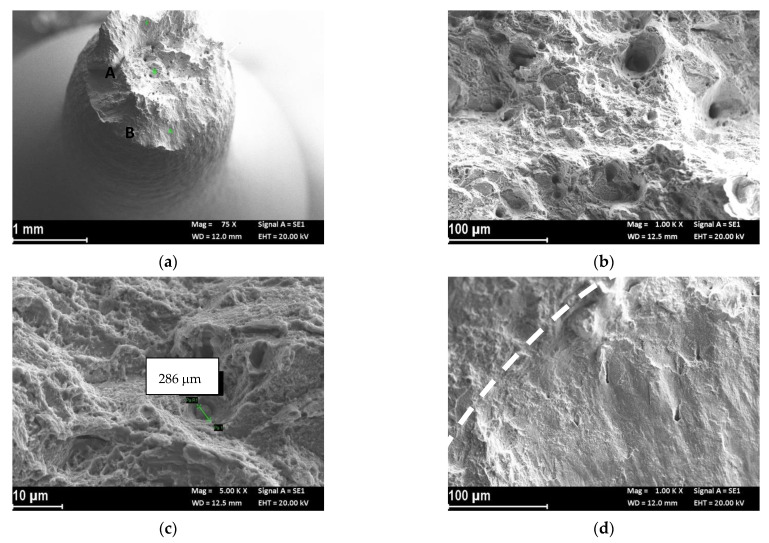
Fractographic analysis of the HT-LPBF specimen after the SSRT test in air: (**a**) macro image; (**b**) close up of the center of the specimen (zone A in (**a**)) with presence of dimples; (**c**) close-up of a large precipitate in (**b**); (**d**) close-up of the border of the specimen (zone B in (**a**)) in which is evident the change of fracture growth in the corresponding edge of the melt pool.

Figure 6a shows the fracture surface of the heat-treated specimens tested in air. The fracture surface had a typical cup and cone shape with a morphology in the center characterized by dimples of very variable dimensions (Figure 6b). Larger dimples were sometimes associated with large-sized precipitates (Figure 6c). The final fracture, also in this case, occurred by sliding by sharing (Figure 6d), with separations between the melt pools (dotted line in Figure 6d).

**Figure 7:**
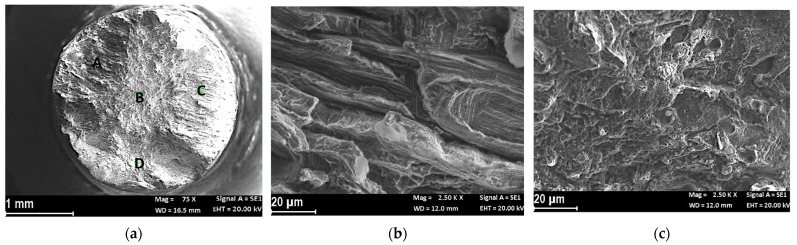
Fractographic analysis of the HT-LPBF specimen after the SSRT test at strain rate 3.15 × 10^−6^ s^−1^ in SOW and cathodic polarization at −1.1 V vs. Ag/AgCl: (**a**) macro image; (**b**) close-up of the trigger of the fracture (zone A of (**a**)—zone C has the same morphology of zone A; (**c**) close-up of the center of the specimen (zone B of (**a**)—a similar morphology is present in zone D of (**a**)).

The fracture surface of the heat-treated specimen at the end of the SSRT test with strain rate 3.15 × 10^−6^ s^−1^ cathodically polarized at −1.1 V vs. Ag/AgCl in SOW was totally flat (Figure 7a); there were two large trigger zones by hydrogen embrittlement (denoted with A in the Figure 7a) with a morphology similar to the UT specimens (Figure 7b), with flat facets arranged in a radial direction, separated by longitudinal microfractures and flat areas of cleavage. Even the center of the specimen appeared with a quasi-cleavage morphology (areas B and C—close-up in Figure 7c), with a sporadic presence of small dimples.

**Figure 8:**
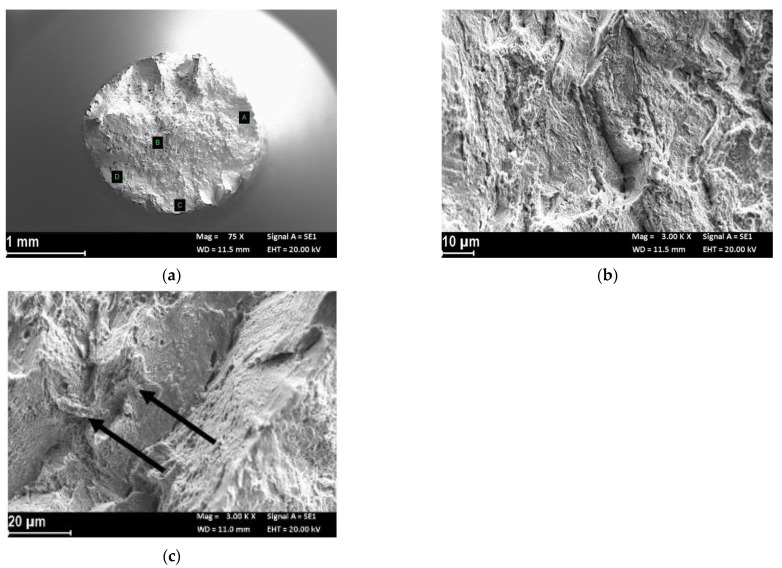
Fractographic analysis of the HT-LPBF specimen after the SSRT test at strain rate 3.15 × 10^−5^ s^−1^ in SOW and cathodic polarization at −1.1 V vs. Ag/AgCl after 16 h of hydrogen pre-charging: (**a**) macro image; (**b**) close up of the trigger of the fracture (zone A in (**a**)); (**c**) close-up of the center of the specimen (zone B in (**a**)), the arrows indicate second phases.

Finally, Figure 8a shows the fracture surface of the specimen preloaded with hydrogen for 16 h and then subjected to an SSRT test at a strain rate of 3.15 × 10^−5^ s^−1^. Contrary to the previous case, the fracture surface showed necking, with small triggers of HE on the lateral surface (zones C and D of Figure 8a) with a mixed morphology with dimples and small areas of quasi-cleavage (Figure 8b). Conversely, the center of the specimen displayed mainly small dimples, but with separations along the longitudinal axis of quasi-cleavage (Figure 8c).

## 4. Discussion

The Alloy 625 obtained by LPBF was not susceptible to chloride stress corrosion cracking within the timeframe tested in neither as-built conditions nor after heat treatment, thus displaying a similar resistance for the same alloy obtained by traditional hot working techniques [41].

The results of the SSRT test in air were in agreement with the tensile results of the alloy published by some of the authors of this paper in other publications [39,42], as well as with the results of Yadroitsev et al. [43]. The ultimate tensile strength of the LBPF alloy was higher than the corresponding as-rolled Alloy 625 [44]. The high tensile properties of these materials derived from the very fine dendritic microstructure, together with high dislocation density owing to the fast solidification and cooling rates of the LPBF process [39]. The large dimples present on the fracture surface could be due to the presence of microvoids generated by gas porosity, un-melted powder or second phases. The brittle fractures’ transgranular cleave-like facets (zone 3 in Figure 3c), were attributed by Brown et al. [45] to the segregation of Nb and Mo in the interdendritic regions. Moreover, the brittle fracture can also derive from the presence of intergranular carbides [42]. Heat treatment at 980 °C tended to increase the ductility and reduce the tensile strength due to the dissolution of the sub-grain structures and mitigation of the dislocations [46].

**Figure 9:**
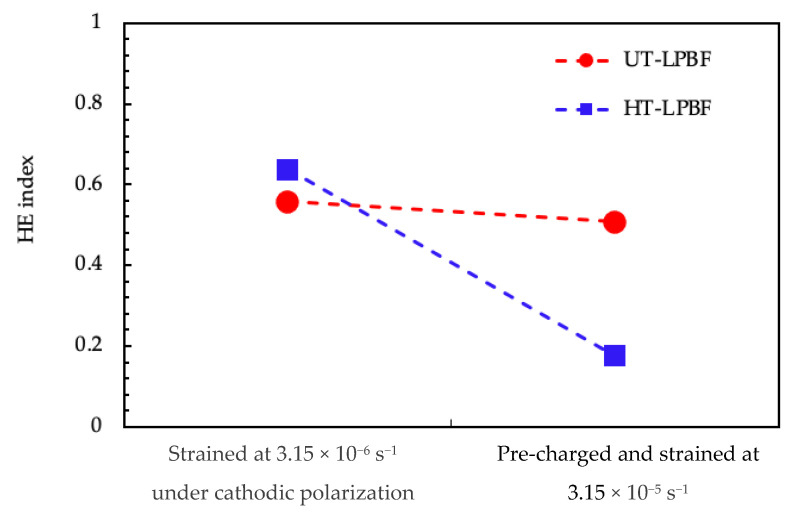
Hydrogen embrittlement index for the LPBF specimens after SSRT tests.

Alloy 625 underwent hydrogen embrittlement in SOW when cathodically polarized, as summarized in Figure 9 where the HE_index_ were reported. However, despite every specimen displaying evidence of hydrogen embrittlement, the highest susceptibility was generally identified for the cathodically polarized samples tested at 3.15 × 10^−6^ s^−1^.

All SSRT tests had the same time of hydrogen charge, and it could be predicted that 16 h of cathodic pre-charging would permit hydrogen to permeate the full thickness of the specimen (3 mm). However, it was seen that the samples were much more resistant to hydrogen embrittlement when tested at the high strain rate. This effect was more pronounced for the HT-LPBF specimen than the UT-LPBF specimens, although more work will be required to understand whether these differences are significant. It is evident that the stress and triaxiality during the cathodic polarization enhanced the hydrogen absorption; this could be due to hydrogen viscous dragging by the dislocation (Cottrell atmosphere type) that enhances the hydrogen mobility [47].

The hydrogen embrittlement susceptibility of nickel and its alloys has been well known for many years [48]; Kane et al. [31] performed constant load tests on cold-deformed Alloy 625 specimens in acid solutions, analysing the effect of cathodic polarization. Failure times decreased from thousands of hours to tens of hours upon application of l0 mA/cm^2^ cathodic current density. Aging at 500 °C decreased the failure time by almost two orders of magnitude regardless of the applied potential. Hicks and Altstetter reported that the Alloy 625 changed its morphology of fracture from microvoid coalescence to isolated (111) faced normal to the tensile axis, separated by tear ridges and microvoids coalescence, as the hydrogen content increased [32].

Several authors have pointed out the role of impurity elements and/or second phase precipitates in HE phenomena. Bruemmer et al. [49] reported that the enrichment of certain metalloid impurity elements at grain boundaries induced intergranular failure of nickel, iron, and several of their alloys in the presence of hydrogen. Latanision and Opperhauser [50] demonstrated that the hydrogen embrittlement of nickel 270 was associated with the enrichment of antimony and tin at grain interfaces, while Berkowitz and Kane [51] and Fiore and Kargol [52] suggested that phosphorus played a part in the hydrogen-induced cracking of a nickel base superalloy. However, the Alloy 625 used in this work had a very low content of these elements, so their effect on the brittle fracture in the presence of hydrogen can be excluded.

Jothi et al. [53] reported that microstructural features such as grain boundaries (GBs) may have promoted faster diffusion of hydrogen due to the locally disordered atomic structure, but also acted as hydrogen trap (or segregation) sites when the probability of atomic hydrogen jumping into GB sites (capture) was greater than that of atomic hydrogen jumping out of GB sites (escape).

Harris et al. [54] reported that the grain boundary diffusion coefficient was higher than the lattice diffusion coefficient. A recent model developed by Jothi et al. [46] evidenced that hydrogen diffusion and segregation in micro-polycrystalline and nano-polycrystalline material between grains and grain boundaries were inhomogeneous and much greater hydrogen concentrations were accumulated at grain boundaries than within grains. Hydrogen diffusion and segregation was greater in nano-polycrystalline material due to the higher density of grain boundaries [41]. It could be postulated that hydrogen diffusion through the UT-LPBF specimens during pre-charge should be more efficient than that on HT-LPBF specimens owing to the finer microstructure. In a previous work by the present authors, it was observed that heat treatment at 980 °C for 32′ dissolved the melt pool microstructure obtained by the LPBF process, producing some niobium carbide formation without appreciable recrystallization [23]. For these reasons, no appreciable differences were observed in the SSRT at 3.15 × 10^−6^ s^−1^ and scathodic polarization. The fractographic analysis evidenced for both the specimens showed similar fracture surfaces. The alternate presence of dimples and brittle area on the fracture surface, evidenced in the fractographic analysis (Figure 4 and Figure 6), seemed to indicate a role of second phases in hydrogen embrittlement. This role is masked in the test at the lower strain rate by the high hydrogen supply due to the continuous straining.

Conversely, second phases could have effects on hydrogen trapping during the pre-charge, and its release during the high strain rate test. The role of second phases in hydrogen diffusion and embrittlement is not well understood: Brass et al. reported that the interaction of hydrogen with dislocations and γ’ precipitates involved both hydrogen induced strengthening but also a glide plane softening associated with a hydrogen induced strain localization [55]. If the hydrogen is charged before the beginning of the mechanical straining, the second phases’ behavior as traps or wells could depend on their size and coherency. It could be hypothesized that in the UT specimens, the small and coherent second phases act as reversible traps during pre-charge, and therefore, assist HE during straining. The heat treatment increases their dimensions, so they become incoherent and trap stronger, that mitigates HE, provided they are not in the cracking path. This effect must be better analyzed, therefore it will be object of future work.

## 5. Conclusions

The paper investigated the stress corrosion cracking and hydrogen embrittlement behavior of Alloy 625 obtained by laser powder bed fusion additive manufacturing technique, in either as-built or after a stress relieved treatment at 980 °C for 32 min. The following conclusions can be drawn:

(1) Irrespective of the heat treatment, LPBF manufactured Alloy 625 was not susceptible to chlorides induced stress corrosion cracking;
(2) The samples underwent hydrogen embrittlement when tested under slow strain rate tests in substitute ocean water when cathodically polarized at −1.1 V vs. Ag/AgCl. The HE effects were evident for the UT specimens both on a very low strain rate and at a higher strain rate on specimens pre-charged with hydrogen;
(3) The HT specimens showed a similar behavior to the UT specimens in tests at the very low strain rate, but a good resistance in tests at the higher strain rate on hydrogen pre-charged specimens;
(4) The different behavior of the heat-treated alloy was explained on the basis of the different microstructure: the very fine sub-grain structures, high concentration of dislocation and second phases increased the hydrogen diffusion and reversible trapping effect in the UT specimens;
(5) It is postulated that the coalescence of precipitates during heat treatment increased their incoherency and made them irreversible traps for hydrogen that mitigated HE effects in the HT specimens.

## Figures and Tables

**Table 1:** Chemical composition of Alloy 625 powder and of the LPBF samples.

Element (%wt)	C	Si	Mn	P	S	Cr	Mo	Ni	Nb	Ti	Al	Co	Ta	Fe	Nb + Ta
LPBF powder	0.013	0.1	0.03	<0.001	0.002	22.8	8.1	Bal	3.66	0.17	<0.01	0.17	0.13	0.43	3.79
LPBF metal	0.01	0.08	0.03	<0.001	0.002	22.4	8.2	Bal	3.73	0.18	<0.01	0.17	0.13	0.45	3.86

**Table 2:** Results of SCC tests conducted in boiling magnesium chlorides at 155 and 170 °C using U-bend specimens after 170 h immersion.

Specimen	Temperature [°C]	Building Direction	Initial Weight * [g]	Final Weight * [g]	ΔM [g]	Crack
U-bend LPBF UT	155	XY	29.80806	29.80671	0.00134	NO
		Z	27.21266	27.20922	0.00344	NO
U-bend LPBF UT	170	XY	27.16713	27.16435	0.00278	NO
			26.90112	26.89606	0.00507	NO
		Z	31.46677	31.46087	0.00591	NO
			31.46682	31.46087	0.00596	NO
U-bend LPBF HT 980 °C	170	XY	27.85226	27.84929	0.00297	NO
			28.27807	28.27352	0.00455	NO
		Z	28.59065	28.58455	0.00610	NO
			28.76252	28.58455	0.17797	NO

* average value on three measures.

**Table 3:** Results of the SSRT tests.

Specimens	Test	Strain Rate (s^−1^)	σ_max_ (MPa)	σ_max env/_ σ_max air_	ε_max_ (%)	RA (%)	HE _index_ (%)
LPBF-UT	Air	3.15 × 10^−6^	1018	-	32	61	-
	SOW E = −1.1 V vs. Ag/AgCl	3.15 × 10^−6^	995	0.98	25	27	56
	16 h pre-charging in SOW E = −1.1 V vs. Ag/AgCl than test in the same solution	3.15 × 10^−5^	985	0.97	26	30	51
LPBF-HT	Air	3.15 × 10^−6^	953	-	41	61	-
	SOW E = −1.1 V vs. Ag/AgCl	3.15 × 10^−6^	894	0.94	19	22	64
	16 h pre-charging in SOW E = −1.1 V vs. Ag/AgCl than test in the same solution	3.15 × 10^−5^	874	0.92	38	50	18

## Data Availability

All data are contained within the article.

## Supplementary Materials

The following are available online at https://www.mdpi.com/article/10.3390/ma14206115/s1, Figure S1. U-bend specimens, Figure S2. direction of printing of U-bend, Figure S3. Cell for SSRT.
