# Supplementary material for: Stress Corrosion Cracking of Additively Manufactured Alloy 625
Source: Materials (Basel). 2021 Oct 15;14(20):6115. doi: 10.3390/ma14206115 (PMC8541440; doi:10.3390/ma14206115)
Supplement: Supplementary file 1 [file materials-14-06115-s001.zip › materials-1348049-supplementary.pdf]

Supplementary materials

Stress Corrosion Cracking of Additively Manufactured Alloy 625

Marina Cabrini [1,2,*], Sergio Lorenzi [1,2], Cristian Testa [1,2], Francesco Carugo [1], Tommaso Pastore [1,2], Diego Manfredi [3], Sara Biamino [3], Giulio Marchese [3], Simone Parizia [3] and Fabio Scenini [4]

1 Department of Engineering and Applied Sciences, School of Engineering, University of Bergamo, 24044 Dalmine, Italy; sergio.lorenzi@unibg.it (S.L.) cristian.testa@guest.unibg.it (C.T.); francesco.carugo@unibg.it (F.C.); tommaso.pastore@unibg.it (T.P.)
2 Research Unit of Bergamo of National Interuniversity Consortium of Materials Science and Technology (INSTM), 24044 Dalmine (BG), Italy
3 Department of Applied Science and Technology, Politecnico di Torino, 10129 Torino, Italy; diego.manfredi@polito.it (D.M.); sara.biamino@polito.it (S.B.); giulio.marchese@polito.it (G.M.); simone.parizia@polito.it (S.P.)
4 Materials Performance Centre and Corrosion and Protection Centre, Department of Materials, University of Manchester, Manchester M13 9PL, UK; fabio.scenini@manchester.ac.uk
* Correspondence: marina.cabrini@unibg.it; Tel.: +39-035-2052316

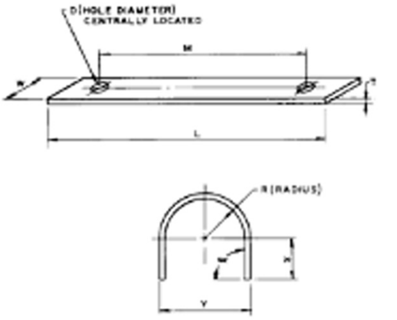

L (mm)	M (mm)	W (mm)	T (mm)	D (mm)
80	50	20	2.5	10
X (mm)	Y (mm)	R (mm)	α (rad)	
32	14	5	1.57	

Figure S1. U-bend specimens.

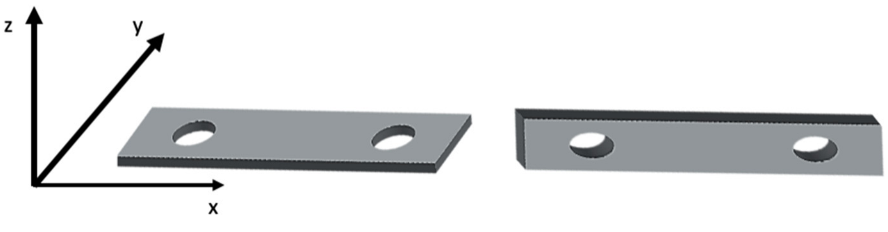

Figure S2. direction of printing of U-bend.

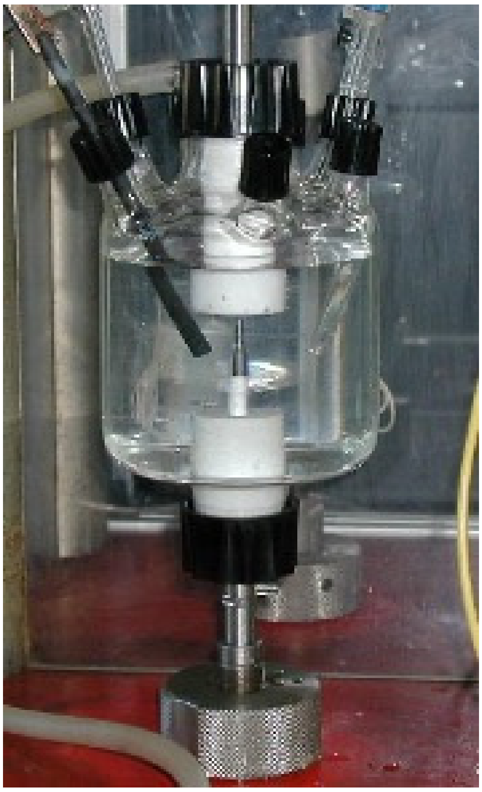

Figure S3. Cell for SSRT.
